# Bystanders Saving Lives with Naloxone: A Scoping Review on Methods to Estimate Overdose Reversals

**DOI:** 10.5811/westjem.18037

**Published:** 2024-05-21

**Authors:** Andrew T. Kinoshita, Soheil Saadat, Bharath Chakravarthy

**Affiliations:** * University of California Irvine, School of Medicine, Irvine, California; † University of California Irvine, School of Medicine, Department of Emergency Medicine, Irvine, California

## Abstract

**Introduction:**

People who use drugs in community settings are at risk of a fatal overdose, which can be mitigated by naloxone administered via bystanders. In this study we sought to investigate methods of estimating and tracking opioid overdose reversals by community members with take-home naloxone (THN) to coalesce possible ways of characterizing THN reach with a metric that is useful for guiding both distribution of naloxone and advocacy of its benefits.

**Methods:**

We conducted a scoping review of published literature on PubMed on August 15, 2022, using PRISMA-ScR protocol, for articles discussing methods to estimate THN reversals in the community. The following search terms were used: *naloxone AND (“take home” OR kit OR “community distribution” OR “naloxone distribution”)*. We used backwards citation searching to potentially find additional studies. Overdose education and naloxone distribution program-based studies that analyzed only single programs were excluded.

**Results:**

The database search captured 614 studies, of which 14 studies were relevant. Backwards citation searching of 765 references did not reveal additional relevant studies. Of the 14 relevant studies, 11 were mathematical models. Ten used Markov models, and one used a system dynamics model. Of the remaining three articles, one was a meta-analysis, and two used spatial analysis. Studies ranged in year of publication from 2013–2022 with mathematical modeling increasing in use over time. Only spatial analysis was used with a focus on characterizing local naloxone use at the level of a specific city.

**Conclusion:**

Of existing methods to estimate bystander administration of THN, mathematical models are most common, particularly Markov models. System dynamics modeling, meta-analysis, and spatial analysis have also been used. All methods are heavily dependent upon overdose education and naloxone distribution program data published in the literature or available as ongoing surveillance data. Overall, there is a paucity of literature describing methods of estimation and even fewer with methods applied to a local focus that would allow for more targeted distribution of naloxone.

Population Health Research Capsule What do we already know about this issue?

*Administration of naloxone mitigates the risk of a fatal overdose in community settings; however, surveillance of community naloxone and its administration is weak.*

What was the research question?

*What methods exist for tracking or estimating opioid overdose reversals by community members with naloxone?*

What was the major finding of the study?

*The scoping review yielded 14 studies: 11 mathematical models, one meta-analysis, and two spatial analyses.*

How does this improve population health?

*Few methods have been published to estimate community naloxone administration; methods must be adapted for local use before informing policy or advocacy.*

## INTRODUCTION

People who use drugs in community settings have the risk of a fatal overdose, which can be mitigated by naloxone administered via bystanders during overdose incidents. Currently, there is some public health infrastructure in place to track naloxone distribution. In California, the Department of Health Care Services (DHCS) acts as a hub for dissemination of naloxone to community-based organizations.^1^ These organizations are, in turn, charged with maintaining distribution and use data. However, the DHCS is not the only distributor of naloxone, nor do programs that distribute naloxone have any way to require individuals to report use. Further, naloxone in Narcan nasal spray form has recently been approved (in March 2023) by the US Food and Drug Administration for over-the-counter (OTC) distribution. Due to this multitude of factors, it is not known how frequently community-distributed naloxone is administered to treat overdose.

While naloxone distribution is an effective, evidence-based intervention, and OTC formulations are approved, there is still pushback against highly visible and available naloxone distribution points from policymakers and community members due to the stigma associated with drug use and, by extension, the legal landscape.^2^^,^^3^ In this study we sought to investigate methods of estimating and tracking opioid overdose reversals by community members with take-home naloxone (THN) to coalesce possible ways of characterizing THN reach with a metric that is useful for guiding both distribution of naloxone and advocacy of its benefits.

## METHODS

With PRISMA-ScR protocol using the PubMed database,^4^ we conducted a scoping review on methods to estimate opioid overdose reversals by community members using THN, before any potential intervention by first responders or clinicians. The database search was followed by backwards citation searching to identify relevant articles omitted in the database search. PubMed, a database provided by the National Center for Biotechnology Information at the US National Library of Medicine, was used for the scoping review due to its coverage of 35 million citations contained within the literature compilations of MEDLINE, PubMed Central, and Bookshelf.^5^

### Search Strategy

We performed a search on August 15, 2022, using PubMed to find articles that discussed surveillance or estimation of THN administration. The search was restricted to articles published in the English language, but it was not restricted by year of publication. The terms used for the search strategy were selected to ensure that relevant studies found in pilot searches were all included. Since there has been an evolving lexicon surrounding “take-home” naloxone, alternative terms had to be included in the search, even though this diluted the proportion of relevant studies in the final search. We used the following search terms: *naloxone AND (“take home” OR kit OR “community distribution” OR “naloxone distribution”)*.

Articles from the PubMed search that discussed THN and were possibly related to surveillance or estimation were sorted into methodology buckets for possible further review based on title and abstract, or review of full articles where uncertainty existed. These methodology buckets included the following: 1) mathematical models; 2) meta-analysis; 3) spatial analysis; 4) other possibly relevant articles; 5) opioid overdose education and naloxone distribution (OEND) program-based studies; and 6) other articles deemed not relevant.

The articles sorted into the first four buckets—mathematical models; meta-analysis; spatial analysis; and other possibly relevant articles—were read in full for confirmation of final inclusion. We excluded from further review bucket 5 (OEND program-based studies) because these studies have straightforward methodology and are already a well-known method of tracking THN administration, which is evidenced by the number of OEND program-based studies (59 studies captured with our database search strategy). These OEND program-based studies are discussed further in the *Discussion* section. After selection of PubMed articles for final inclusion, we performed backwards citation searching on these articles using titles, with abstracts as needed. The full text of possibly relevant articles was reviewed for final inclusion.

### Data Extraction and Synthesis

We extracted the following data using a standardized table: method (bucket); model type; data sources; location (country, location – community); and funding sources. Method corresponded to the bucket categories discussed above. Model type was relevant for studies in bucket 1 (mathematical models), and the recorded model type was based on how authors self-described their studies. These self-descriptions for mathematical models included Markov modeling and system dynamics modeling. Data was synthesized through concept mapping.

## RESULTS

**Figure 1.**
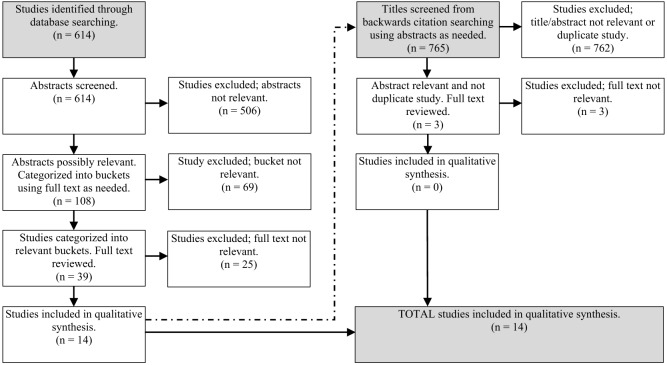
PRISMA flowchart.

The database search resulted in the capture of 614 studies. Of these, 108 studies were marked as possibly relevant based on titles or abstracts discussing THN programs, surveillance, or estimation. Using full articles as needed, 39 studies were categorized into buckets of interest (1–4). Following categorization, full article review resulted in 14 articles for final inclusion. Backwards citation searching of the 765 references contained within the 14 articles resulted in three articles for full review. All three were excluded from final analysis leaving 14 articles for final inclusion. These 14 articles were from buckets 1–3. Figure 1 presents a flowchart of the captures and the review of literature.

### Study Characteristics

The included studies varied in their objectives. Developing a way to identify how much naloxone was administered by bystanders was often a contributor to the overall goals of the studies instead of the primary objective. This section presents a synthesis of study objectives and the methods employed to surveil or estimate community naloxone use. The Table presents an overview of the studies by method.

**Table.** Study characteristics by method.

Method (bucket)	Model type	First author	Year	Data sources	Location country	Location community	Funding sources
Mathematical models	Markov model	Acharya M	2020	Literature, Surveillance data, Assumption	US	US	Not reported
		Coffin PO	2022	Literature, Assumption	US	US	National Institutes of Health
		Coffin PO	2013	Literature, Expert input, Assumption	US	US	National Institute of Allergy and Infectious Diseases (National Institutes of Health)
		Coffin PO	2013	Literature, Assumption	Russia	Russia	Open Society Foundation
		Irvine MA	2018	Surveillance data, Literature, Expert input, Assumption	Canada	British Columbia	Canadian Institutes of Health Research, Natural Science and Engineering Research Council of Canada
		Irvine MA	2019	Surveillance data, Literature, Expert input, Assumption	Canada	British Columbia	British Columbia Government, Canadian Institutes of Health Research, Natural Science and Engineering Research Council of Canada, Michael Smith Foundation for Health Research, National Institutes of Health
		Irvine MA	2022	Literature, Modified-Delphi panel	US	US	National Institute on Drug Abuse (National Institutes of Health)
		Langham S	2018	Literature, Assumption	UK	UK	Mundipharma International Ltd.
		Linas BP	2021	Surveillance data, Literature, Assumption	US	Rural, urban Massachusetts	National Institute on Drug Abuse (National Institutes of Health)
		Uyei J	2017	Surveillance data, Literature, Assumption	US	Connecticut	Connecticut Department of Public Health, National Institute of Mental Health (National Institutes of Health)
	System dynamics model	Stringfellow EJ	2022	Surveillance data, Literature, Expert input, Assumption	US	US	US Food and Drug Administration
Meta-analysis		McAuley A	2015	OEND program studies	Canada, UK, US	n/a	National Health Service Scotland
Spatial analysis		Rowe C	2016	Surveillance data	US	San Francisco	National Institute on Drug Abuse (National Institutes of Health)
		Yi G	2022	Surveillance data	US	Baltimore	Not reported

### Mathematical Models

Of the 14 studies, 11 employed mathematical models. Of these, 10 used Markov models and were published between 2012–2022. Markov models define several non-overlapping statuses (ie, chronic opioid use, cessation of opioid use, overdosing, dead) and represent each individual within a simulated population as a member of one of the statuses.^6^ Individuals transition from one state to another, not necessarily linearly, based on probability parameters that represent change in individual statuses over time. This means that model output of any prior or subsequent population distribution within the system can be derived from any given population distribution. The one remaining mathematical modeling study used a system dynamics model and was published in 2022. System dynamics modeling represents different variables (ie, population, treatment availability, overdose deaths) within a system and the relationships between them, factoring in temporal delay as appropriate.^7^ This means that the model output of any subsequent population distribution within the system may be based on both the given population distribution and the changes preceding the given population distribution.

Studies employing mathematical models varied in their primary objectives. Five of the studies employing Markov models were designed to evaluate the cost effectiveness of naloxone distribution. Four of these cost-effectiveness studies use variations of the same Markov model, which was originally developed in 2013 by Coffin and Sullivan, who authored two of the four articles.^8^^–^^11^ The one remaining cost-effectiveness study, by Uyei et al, was unique in that it also investigated naloxone distribution in conjunction with other interventions, including pre-exposure prophylaxis for HIV prevention.^12^

Of the remaining five Markov model studies, all modeled the effects of naloxone distribution on opioid overdose death rates. Coffin et al (2022) modeled the US population using the Markov model developed previously by Coffin and Sullivan in 2013.^13^ Irvine et al (2018) and Irvine et al (2019) modeled the population of British Columbia using a model developed by Irvine et al in 2018.^14^^,^^15^ Irvine et al (2022) modeled the US population, and Linas et al (2021) modeled urban and rural Massachusetts populations also using the 2018 Irvine et al model.^16^^,^^17^

The one study using a system dynamics model was conducted by Stringfellow et al in 2022 and investigated the effects of different interventions, including naloxone distribution, on opioid overdose death rates.^18^

Mathematical models employed various data sources to inform the parameters used. These sources included parameters from published literature and surveillance data (ie, public health department records, coroner reports, insurance claims). When sources of data were not available, authors used their own assumptions or expert input, including a modified-Delphi panel in the 2022 Irvine et al study.^16^ The studies do not apply the mathematical models to any specific cities or smaller communities, although the 2021 Linas et al study models a generalized rural city and a generalized urban city in Massachusetts.^17^ Adopting the mathematical models employed in these studies to estimate bystander naloxone administration in a particular community of interest would require the input of local parameters, which could be an intensive effort if surveillance infrastructure is not established.

### Meta-analysis

One study by McAuley et al, published in 2015, consisted of a meta-analysis of nine OEND program studies, synthesizing their outcomes and accounting for participants lost to follow-up to report the proportion of naloxone kits that are likely to be used in the first three months after distribution.^19^ The studies that comprised the meta-analysis were from Canada, the United Kingdom, and the US. Adopting a meta-analysis methodology to estimate bystander naloxone administration in a particular community of interest would involve synthesizing data from OEND programs in the community.

### Spatial Analysis

Two studies, by Rowe et al (2016) and Yi et al (2022), used geographic system information (GSI) mapping technology to conduct spatial analysis of naloxone overdose incidents. The studies determined the relationship between proximity of the census tract in which naloxone was administered and the nearest naloxone distribution site.^20^^,^^21^ Rowe et al conducted an analysis of San Francisco, California, and Yi et al conducted an analysis of Baltimore, Maryland. Surveillance data was used to establish this relationship. The GSI mapping and spatial analysis methodology used in these studies could be adopted in other jurisdictions to estimate bystander naloxone administration in a particular neighborhood of interest based in part on distance from naloxone distribution points.

## DISCUSSION

### Limited Methods to Estimate Take-home Naloxone Use

The limited number of studies captured in this scoping review evidences the lack of surveillance and estimation methods for the administration of THN, outside of OEND program records based on self-reports. Of the methods used, mathematical modeling and meta-analysis provided direct estimations of the proportion of distributed naloxone administered; however, both methods were applied only over large geographic areas (entire countries, states or provinces, amalgamating different cities around the globe) or theoretical cities representing a large geographic area (“urban city of Massachusetts”).

**Figure 2.**
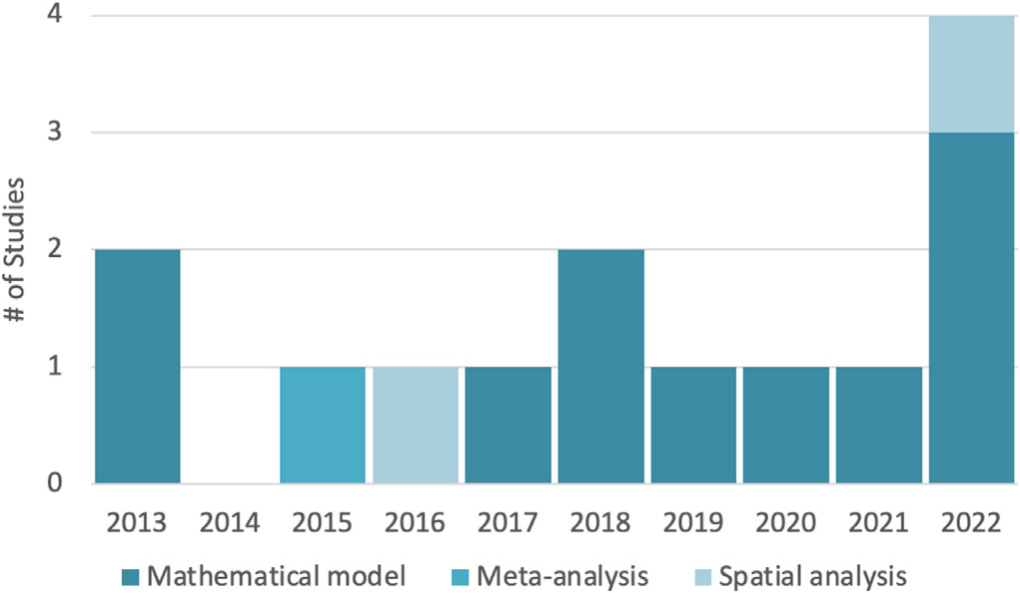
Methods used over time.

Mathematical modeling was the most popular form of estimating administration of naloxone by community members. Further, the popularity of modeling increased relative to the other methods. While making up 79% of study methodologies found overall, it comprises 89% of studies in the five years from 2018–2022, as shown in Figure 2. Reasons for the popularity of mathematical models may be convenience, including the use of expert input and assumptions for unknown parameters, and the ability to tailor models to different geographic areas by adjusting parameters. Nine of the 11 modeling studies used one of two model bases, Coffin and Sullivan (2013) and Irvine et al (2018).^9^^,^^14^

The relative disuse of meta-analysis may be explained by the lower practical value of naloxone administration data averaged over multiple locations, as opposed to applying local data to inform program growth and gauge impact. Meta-analysis of naloxone use in other communities may be informative in jurisdictions lacking their own surveillance data, but care must be exercised in selecting which communities and programs to use as references. The spread of OEND programs, however, may provide an opportunity for more applicable comparisons. Further, large proportions of follow-up loss are evidenced in some OEND programs, adding uncertainty to meta-analysis results; three of the nine OEND programs that McAuley et al (2015) used in their analysis had three-month follow-up rates of less than 70% (eg, 34%, 30%, 23%).^19^

Spatial analysis yielded a relationship between naloxone administration and distance from naloxone distribution point. Both studies included in this scoping review (Rowe et al 2016, and Yi et al 2022) were reliant upon self-reported data from OEND programs. This data, which is needed to construct a GSI map, may be useful for identifying geographic areas for intervention but may be less useful for extrapolation to unreported THN use. Further, only the study by Yi et al (2022) characterized the relationship between probability of bystander naloxone administration at an overdose and distance from distribution point.^21^ Rowe et al (2016) instead reported total number of administration events as a function of distance, further limiting external validity of the results.^20^

### Opioid Education and Naloxone Distribution Programs

While we excluded individual OEND program-based studies from this scoping review, they are important for discussion and comprised 59 of the captured articles in the systematic search. Data from these programs, whether or not published in peer-reviewed journals, is the foundation for the parameters in mathematical models, the component studies of meta-analysis, and the location data for spatial analysis. The accuracy of all methods to estimate naloxone administration by bystanders wraps back around to the quality of self-reported data from OEND programs. When estimations of THN use are put forward to inform policy, the methods behind the estimate must be justifiably better than local OEND data, if available. Amalgamated data provided by government institutions and national coalitions may also be available but will lack local specificity.^22^^,^^23^

## LIMITATIONS

There are limitations to this scoping review and its applicability. In our study we did not attempt to include methods published in the gray literature in our initial search strategy. This limitation was addressed in part through informal preliminary searches, correspondence with public health personnel at the California Department of Public Health and the CA Bridge program, and citation searching. Further, it was not expected that methods for estimation of bystander naloxone use would exist without being published in peer-reviewed journals.

A related limitation of this study is that the initial search for relevant articles was limited to the PubMed database. This decision was based on the PubMed search terms comprehensively capturing all studies identified by previous informal preliminary searches and correspondence with public health personnel. Additionally, the search strategy attempted to capture any potentially missed literature through backwards citation searching, and the absence of any new relevant articles supported the parameters of the initial search.

Another limitation to this scoping review is that it did not attempt to ascertain the comparative value of methods used in estimating bystander naloxone use. It is possible that preferred methods for determining bystander naloxone use will be dependent upon intended use of the analysis and preference for risk. Methods highly influenced by OEND program data will inherently provide underestimation, while others may cause overestimation. Finally, the environment surrounding harm reduction is constantly changing. The recent approval of OTC naloxone is a new policy that the studies captured in our review do not address.

## CONCLUSION

The present scoping review describes the available methods for estimating bystander administration of naloxone. Mathematical models, particularly Markov models, are most common. System dynamics modeling, meta-analysis, and spatial analysis have also been used. All methods are heavily dependent upon OEND program data published in the literature or available as ongoing surveillance data. Overall, there is a paucity of literature describing methods of estimation, and of these few have been applied with a local focus. This is of concern as harm reduction is still regarded with stigma. Further, even as naloxone distribution becomes more normalized, both politically and socially, effective distribution will remain important in a landscape of funding and resource scarcity with complementary interventions and competing policy priorities.

## References

[1] California Department of Health Care Services . Naloxone distribution project. 2023. Available at: https://www.dhcs.ca.gov/individuals/Pages/Naloxone_Distribution_Project.aspx. Accessed March 22, 2023.

[2] Martignetti L Sun W . Perspectives of stakeholders of equitable access to community naloxone programs: a literature review. Cureus. 2022;14(1):e21461. 35223245 10.7759/cureus.21461 PMC8858082

[3] Carroll JJ Green TC Noonan RK . Evidence-based strategies for preventing opioid overdose: what’s working in the United States. *CDC*. 2018. Available at: http://www.cdc.gov/drugoverdose/pdf/pubs/2018-evidence-based-strategies.pdf. Accessed March 22, 2023.

[4] Tricco AC Lillie E Zarin W et al . PRISMA extension for scoping reviews (PRISMA-ScR): checklist and explanation. Ann Intern Med. 2018;169(7):467–73. 30178033 10.7326/M18-0850

[5] National Library of Medicine . PubMed overview. Available at: https://pubmed.ncbi.nlm.nih.gov/about/. Accessed March 29, 2023.

[6] Sonnenberg FA Beck JR . Markov models in medical decision making: a practical guide. Med Decis Making. 1993;13(4):322–38. 8246705 10.1177/0272989X9301300409

[7] Wang Y Hu B Zhang Y et al . Applications of system dynamics models in chronic disease prevention: a systematic review. Prev Chronic Dis. 2021;18:E103. 34941481 10.5888/pcd18.210175 PMC8718124

[8] Acharya M Chopra D Hayes CJ et al . Cost-effectiveness of intranasal naloxone distribution to high-risk prescription opioid users. Value Health. 2020;23(4):451–60. 32327162 10.1016/j.jval.2019.12.002

[9] Coffin PO Sullivan SD . Cost-effectiveness of distributing naloxone to heroin users for lay overdose reversal. Ann Intern Med. 2013;158(1):1–9. 23277895 10.7326/0003-4819-158-1-201301010-00003

[10] Coffin PO Sullivan SD . Cost-effectiveness of distributing naloxone to heroin users for lay overdose reversal in Russian cities. J Med Econ. 2013;16(8):1051–60. 23730942 10.3111/13696998.2013.811080

[11] Langham S Wright A Kenworthy J et al . Cost-effectiveness of take-home naloxone for the prevention of overdose fatalities among heroin users in the United Kingdom. Value Health. 2018;21(4):407–15. 29680097 10.1016/j.jval.2017.07.014

[12] Uyei J Fiellin DA Buchelli M et al . Effects of naloxone distribution alone or in combination with addiction treatment with or without pre-exposure prophylaxis for HIV prevention in people who inject drugs: a cost-effectiveness modelling study. Lancet Public Health. 2017;2(3):e133–40. 29253386 10.1016/S2468-2667(17)30006-3

[13] Coffin PO Maya S Kahn JG . Modeling of overdose and naloxone distribution in the setting of fentanyl compared to heroin. Drug Alcohol Depend. 2022;236:109478. 35588609 10.1016/j.drugalcdep.2022.109478 PMC9235402

[14] Irvine MA Buxton JA Otterstatter M et al . Distribution of take-home opioid antagonist kits during a synthetic opioid epidemic in British Columbia, Canada: a modelling study. Lancet Public Health. 2018;3(5):e218–25. 29678561 10.1016/S2468-2667(18)30044-6

[15] Irvine MA Kuo M Buxton JA et al . Modelling the combined impact of interventions in averting deaths during a synthetic-opioid overdose epidemic. Addiction. 2019;114(9):1602–13. 31166621 10.1111/add.14664 PMC6684858

[16] Irvine MA Oller D Boggis J et al . Estimating naloxone need in the USA across fentanyl, heroin, and prescription opioid epidemics: a modelling study. Lancet Public Health. 2022;7(3):e210–8. 35151372 10.1016/S2468-2667(21)00304-2 PMC10937095

[17] Linas BP Savinkina A Madushani RWMA et al . Projected estimates of opioid mortality after community-level interventions. JAMA Netw Open. 2021;4(2):e2037259. 33587136 10.1001/jamanetworkopen.2020.37259 PMC7885041

[18] Stringfellow EJ Lim TY Humphreys K et al . Reducing opioid use disorder and overdose deaths in the United States: a dynamic modeling analysis. Sci Adv. 2022;8(25):eabm8147. 35749492 10.1126/sciadv.abm8147 PMC9232111

[19] McAuley A Aucott L Matheson C . Exploring the life-saving potential of naloxone: a systematic review and descriptive meta-analysis of take home naloxone (THN) programmes for opioid users. Int J Drug Policy. 2015;26(12):1183–8. 26508033 10.1016/j.drugpo.2015.09.011

[20] Rowe C Santos GM Vittinghoff E et al . Neighborhood-level and spatial characteristics associated with lay naloxone reversal events and opioid overdose deaths. J Urban Health. 2016;93(1):117–30. 26800987 10.1007/s11524-015-0023-8 PMC4794468

[21] Yi G Dayton L Uzzi M et al . Spatial and neighborhood-level correlates of lay naloxone reversal events and service availability. Int J Drug Policy. 2022;106:103739. 35691087 10.1016/j.drugpo.2022.103739

[22] Wheeler E Davidson PJ Jones TS et al . Community-based opioid overdose prevention programs providing naloxone — United States, 2010. MMWR Morb Mortal Wkly Rep. 2012;61(6):101–5. 22337174 PMC4378715

[23] Wheeler E Jones TS Gilbert MK et al . Opioid overdose prevention programs providing naloxone to laypersons — United States, 2014. Morb Mortal Wkly Rep. 2015;64(23):631–5. PMC4584734 26086633

